# A survey dataset on determinants of administrative corruption

**DOI:** 10.1016/j.dib.2019.104820

**Published:** 2019-11-16

**Authors:** Salem A. Al-Jundi

**Affiliations:** College of Business, Al Ain University, P.O. Box 64141, Al Ain, United Arab Emirates

**Keywords:** Administrative corruption, Culture, Organizational culture, Political instability, Institutional weakness, Social class

## Abstract

This data article describes a survey dataset on administrative corruption and its main determinants (culture, organizational culture, political instability, and institutional weakness) in addition to social class. Prior research was consulted to determine indicators of the constructs of administrative corruption. Each construct has four reflective indicators while social class has formative variables. All items are measured using a seven-point Likert scale and semantic differential scale types. Through Google Form, I collected 677 responses that reflect the perspective of the general public in Basra, Iraq. The paper shows how to build observed indicators for administrative corruption and its main causes, and a summary of the raw data. The dataset can be reused by other researchers and easily downloaded from the Mendeley Data repository (https://doi.org/10.17632/xh22fsmvmc.2). While the dataset is prepared for building and testing a model using a structural equation modeling approach, it sheds light on the debate regarding an increasingly important topic internationally, and especially in Middle Eastern countries, such as Iraq, that experience high rates of corruption and political instability.

**Table:** Specifications Table

Subject	Economics
Specific subject area	Economic Development, Public Economics
Type of data	Tables Excel sheets
How data were acquired	Based on prior research, four observed indicators are derived for each construct (administrative corruption, culture, organizational culture, political instability, institutional weakness, and social class). All variables are reflective except for social class which is formative. Google Forms was used to administer an e-survey to collect information about these indicators. 677 responses were collected that reflect the perspective of the general public in Basra, Iraq. The questionnaire was translated into Arabic since Iraqis are Arabic native speakers and English is not common there. Check the Arabic version in the link: https://forms.gle/GTcHnrXGNMLH3xS48 The survey dataset can be viewed at https://doi.org/10.17632/xh22fsmvmc.2, while Table 1, Table 2, Table 3, Table 4, Table 5, Table 6 underneath represents the survey in details.
Data format	Raw Excel sheet Codes of observed variables
Parameters for data collection	All items are measured using a seven-point Likert scale and semantic differential scale types. The format of the Likert scale is 1: strongly disagree; 2: disagree; 3: somewhat disagree; 4: neither agree nor disagree; 5: somewhat agree; 6: agree; and 7: strongly agree. The semantic differential scale follows the same pattern.
Description of data collection	The measured items under consideration were translated into Arabic and inserted into Google Forms as an e-survey. Students, employees, and academics from the University of Basra, Iraq, were contacted first. Social media was then used to reach members of the public who were well versed in digital communication in Basra, Iraq. I aimed to reach 1,000 people from different social classes; however, I collected responses from only 715 participants. Of these, 38 responses were deleted because the respondents had not taken the survey seriously and the standard deviation (SD) for answering all questions was close to zero. The final sample thus comprised 677 responses. The process of collecting the data took three months, from August to October 2017.
Data source location	University of Basrah Basrah Iraq Latitude: 30° 30′ 30.67″ N Longitude: 47° 46′ 49.44″ E
Data accessibility	Researchers can easily download the full raw dataset of the survey due to the details here. Repository name: Mendeley Data Data identification number: https://doi.org/10.17632/xh22fsmvmc.2 Direct URL to data: https://data.mendeley.com/datasets/xh22fsmvmc/2 The data is available at: S. A. Al-Jundi, Corruption dataset, Mendeley Data, v2, 2018. https://doi.org/10.17632/xh22fsmvmc.2, [1].
Related research article	S. A. Al-Jundi, A. Shuhaiber, S. S. Al-Emara, The effect of culture and organizational culture on administrative corruption, Int. J. Econ. Bus. Res. 18(4) (2019) 436–451. https://doi.org/10.1504/IJEBR.2019.103096, [2].

**Table:** 

**Value of the Data** • The dataset which shows the attitude of the public is useful to researchers around the world, and specifically in countries experiencing high levels of corruption. Additionally, the data can be used by policy makers in developing countries. • The dataset provides novel and rich data on observed indicators of corruption and its main determinants. The data, along with its codes, are ready to be analyzed using a partial least squares/structural equation modeling approach. • The dataset can be easily downloaded. Researchers can use the same measurement items to collect data for other countries and compare with the current experience of people in Iraq.

## 1 Data

A survey on administrative corruption was conducted to study the main determinants of corruption: culture, organizational culture, political instability, and institutional weakness and social class. Literature was consulted to determine indicators of the constructs of administrative corruption. Each construct has four reflective indicators while social class has formative variables. All items are measured using a seven-point Likert scale and semantic differential scale types. Through Google Forms, I collected 677 responses that reflect the perspective of the general public in Basra, Iraq. The dataset can be reused by other researchers and can be easily downloaded from the Mendeley Data repository [1]. While the dataset is prepared for building and testing a model using a structural equation modeling approach, it sheds light on the debate regarding an increasingly important topic internationally, and especially in Middle Eastern countries such as Iraq, which experience high rates of corruption and political instability.

Literature in the field of corruption does not widely suggest using observed variables to measure corruption and its main latent constructs. However, prior research has helped, even indirectly, to build such indicators. Administrative corruption occurs when public employees, regardless of their level, abuse their power in public organizations for their personal benefit through interactions with citizens who are trying to access basic public goods or services. Bribery is the most common practice of corruption. Public employees facilitate their services under a condition of paying bribes. Embezzlement and fraud are used to take funds and goods from organizations. Collusion with the private sector is seen in contracts between construction companies and public entities or when private sector companies seek license. Nepotism is widespread it comes to appointment in the government. We call such activities corruption since public employees breach governmental rules and laws [3]. Table 1 shows the reflective observed indicators of administrative corruption and the mean score of the sample of 677 responses from the public in Basra, Iraq, in addition to the sample SD. The responses of 677 persons are scattered around the agreement on the pervasiveness of corruption.

**Table 1:** Measurable variables of administrative corruption.

Codes	Items	Scale	Mean	SD
CO1	Bribery has become one of the manifestations of everyday work in public organizations.	From 1: strongly disagree to 7: strongly agree	5.65	1.71
CO2	Embezzlement is a widespread phenomenon among public employees.	From 1: strongly disagree to 7: strongly agree	5.37	1.70
CO3	There is collusion between government departments and construction companies executing government projects.	From 1: strongly disagree to 7: strongly agree	5.79	1.55
CO4	There is a widespread phenomenon of nepotism in the administrative work of public organizations.	From 1: strongly disagree to 7: strongly agree	5.72	1.66

Societal culture is represented by values, beliefs, attitudes, and norms. Culture affects practices among the public. If corruption in widespread, people will adopt negative values and accept corrupt practices. It seems that religious and national deterrents are not strong enough to prevent people from being involved in corrupt practices. It becomes justifiable to breach rules and laws such as traffic and municipal laws, and people pretend to be ill at work, for instance. In turn, corruption can be culturally justified since people show apathy and tolerance toward corruption. They do not strongly reject the practices of corruption and they agree to pay bribes. Corrupt public employees are basically greedy as greediness is a feature of being human [4]. Table 2 displays the reflective measurable variables of corrupt culture and the sample mean and SD. The public admitted that the societal culture in the context of Iraq is corrupt.

**Table 2:** Measurable variables of a corrupt culture.

Codes	Items	Scale	Mean	SD
C1	People's reactions to corruption are weak.	From 1: strongly disagree to 7: strongly agree	5.33	2.24
C2	Many people break laws; for instance, traffic and municipal laws.	From 1: strongly disagree to 7: strongly agree	5.40	2.10
C3	Religious deterrence is no longer an effective factor in preventing state officials from practicing administrative corruption.	From 1: strongly disagree to 7: strongly agree	5.61	1.83
C4	Corrupt people are characterized by greed.	From 1: strongly disagree to 7: strongly agree	6.23	1.55

In the absence of strategic management, the organizational culture in public organizations is weak. The societal culture forms the basis for formulating the organizational culture. People are more honest when others deal with them with full honesty. Schools and universities do not teach their students anti-corruption practices and do not prepare their audience to build and accept a proper code of conduct in their future workplace. Public employees learn greediness from their society. Additionally, they become corrupt only when they acquire power in public entities. Clients and administrators hold the same set of values; the former accept paying bribes occasionally and the latter sometimes receive commissions on facilitating access to public services. Weak organizational culture becomes corrupt and leads to corruption. Public employees do not comply with a code of conduct since there are no codes or leaders themselves break the codes. They are not satisfied with salary scales, promotion schemes, and training opportunities. There is a huge gap between growing demands on public services, such as education, health, and infrastructure, and the available resources, which are limited and misallocated. For this reason, people are not satisfied with the quality of public services [5]. Table 3 presents the reflective measurement items of weak organizational culture and the sample mean and SD. The data reveals that organizational culture is weak as interpreted by the public in Basra, Iraq.

**Table 3:** Measurable variables of a weak organizational culture.

Codes	Items	Scale	Mean	SD
A1	The culture of public organizations has become corrupt.	From 1: very clean to 7: very corrupt	6.01	1.42
A2	The failure of public employees to comply with a code of conduct is a cause of administrative corruption.	From 1: strongly disagree to 7: strongly agree	5.69	1.64
A3	The feeling of dissatisfaction among public employees makes them accept administrative corruption.	From 1: strongly disagree to 7: strongly agree	4.70	2.05
A4	How satisfied are you with the services of public organizations?	From 1: very satisfied to 7: not at all satisfied	5.82	1.78

Political instability positively influences administrative corruption. It leads the government to become short-sighted, ignoring a long-term view. Political parties involved in politics concentrate on dealing with conflicts or even civil wars and hence undervalue infrastructure development and anti-corruption programs. Corruption adversely affects investment in countries associated with high levels of corruption. Political instability is reflected in the inadequacy of legislative bodies in inspecting corruption and the government losing its will to fight corruption. Disputes and sectarian division between parties and powerful groups strongly weaken policies against corruption. Judicial inefficiency is an indicator of instability. The parliament becomes unable to fight corruption because of conflicts among political groups. The local and federal government cannot determine their priorities, resulting in weak performance [6].

The US led alliance disbanded Iraqi military forces after the fall of Saddam Hussein's dictatorial regime in 2003. This led to a weakened government and the rise of extremist militias. The government later suffered from sectarian divisions, and jihadist and other militias established by political parties. The local and federal governments spent their effort to deal with sectarian tensions, tribal fighting and terrorism. Their performance became increasingly poor in the field of public services. The government failed to modernize and equip their organizations with technologies. Thus, corruption became widespread at all levels, from small taxation offices to the Ministry of Defense. Table 4 exhibits the reflective observed indicators of political instability and the mean of the sample of 677 responses of the public in Basra, Iraq, and the sample SD. Political instability causes corruption, as clearly stated by the public in Iraq.

**Table 4:** Measurable variables of political instability.

Codes	Items	Scale	Mean	SD
P1	Parliament is weak when it comes to investigating the problems of administrative corruption.	From 1: very strong to 7: very weak	6.31	1.51
P2	There is no real political will to fight administrative corruption.	From 1: strongly disagree to 7: strongly agree	6.03	1.63
P3	Conflict between parties and sectarian groups has weakened the campaign against administrative corruption.	From 1: strongly disagree to 7: strongly agree	6.09	1.60
P4	How acceptable is the performance of the local and federal governments?	From 1: very acceptable to 7: not at all acceptable	5.58	1.74

Institutional weakness and bureaucracy facilitate corrupt practices in governmental entities in developing countries since complexity of procedures and the absence of information technologies make accessing public services a difficult and lengthy process [3]. The lack of proper accountability leads to misuse of public funds for personal benefits. Low compensation and job dissatisfaction encourage public employees to be corrupt. The cost of accepting bribes becomes low. Appointments in public organizations depend on nepotism and bribery rather than on fair process and qualifications. Law enforcement agencies exhibit misbehavior because of a lack of control mechanisms, including judicial oversight, codes of conduct, and the absence of proper managerial strategies in most organizations. In addition, corrupt employees become a symbol for other employees [7]. Table 5 depicts the reflective measurable indicators of institutional weakness and the mean and the sample SD. The public agree that the structure regarding administrative work in public organizations is weak.

**Table 5:** Measurable variables of institutional weakness.

Codes	Items	Scale	Mean	SD
W1	The administrative work of public organizations is characterized by bureaucracy and complexity of procedures.	From 1: very flexible to 7: very bureaucratic	5.79	1.64
W2	Appointments in public organizations depend more on nepotism than on qualifications.	From 1: strongly disagree to 7: strongly agree	6.16	1.56
W3	There is no accountability of employees for misuse of state funds.	From 1: strongly disagree to 7: strongly agree	5.74	1.66
W4	The use of computers and the Internet in the administrative work of state departments reduces administrative corruption.	From 1: strongly disagree to 7: strongly agree	4.98	1.90

Social class can be tracked using objective measurements (income, wealth, and education) and subjective assessments, such as perceived relative social class rank [8]. Even though corruption is pervasive among all social strata, higher social classes contain relatively more powerful people. They are aware of rules and rights, and can strongly express their opinions. However, lower social classes are made up of people with weaker power and awareness. They tend to tolerate and accept corrupt practices to gain access to public services. If we apply this on a macro level, developed countries enjoy lower levels of corruption compared to developing countries. I asked the respondents about their highest educational level, monthly household income, and the size of their residence as a proxy for their wealth. The participants voluntarily and freely recorded the rank of social class to which they belonged. Table 6 characterizes the formative observed indicators of social class and the mean of the sample of 677 responses, which reflects the attitude of the public in Basra, and the sample SD. Participants belong mostly to middle classes.

**Table 6:** Measurable variables of social class.

Codes	Items	Scale	Mean	SD
S1	What is your highest educational level?	From 1: less than elementary school certificate to 7: PhD	4.57	1.42
S2	What is your monthly household income?	From 1: less than US$400 to 7: more than US$1400	3.81	2.04
S3	How big is your house?	From 1: rent a one-room house to 7: own a five-room house or bigger	4.50	1.86
S4	If we divide society into seven social strata, to which class do you belong?	From 1: lowest to 7: highest	4.19	1.38

The current data article focuses on describing the dataset only. The researcher along with his colleagues applied a partial least squares/structural equation modeling approach to analyze the relationship between stated constructs. We find that the weak organizational culture mediates the effect of corrupt culture on administrative corruption. The higher level of social class strengthens the relationship between culture and corruption [2]. On another research, we conclude that institutional weakness mediates the impact of political instability on corruption, and also mediates the impact of weak organizational culture on corruption [9].

## 2 Experimental design, materials, and methods

The latent constructs can be measured using items as seen in Table 1, Table 2, Table 3, Table 4, Table 5, Table 6. To capture the perspective of the general public in Basrah, the observed variables were translated into Arabic since the majority of people in Iraq are Arabic native speakers and are mostly unfamiliar with English. Google Forms hosted the questionnaire as an e-survey (https://forms.gle/GTcHnrXGNMLH3xS48). Once again, the questionnaire was in Arabic to match the Iraqis mother tongue. It is a privilege of this practice to introduce the attitude of the public, who are not familiar with English, to the international scholars. I invited around 20 close friends and relatives to fill out the survey via email. The questions themselves, and the results, were discussed with five faculty members in the College of Administration and Economics, University of Basrah. Based on these discussions, I made slight changes to the first version of the questionnaire to be consistent with the culture in Iraq and how Iraqis view the problem. The survey thus became much more understandable for all educational backgrounds.

To capture the perspective of the general public in Basrah about administrative corruption and its main causes, I invited university students, administrative staff, and faculty members to fill in the survey. I then encouraged them to help me by inviting their relatives and friends who are Basrah citizens. I also invited my friends via social media such as Facebook, WhatsApp, and Instagram. I used email to contact most of my friends from the Basrah University and the wider community to request they fill in the online survey. Participants could not submit their responses unless they answered all questions. I used my friends and relatives to build a network for reaching the general public till I successfully contacted more than 700 persons.

I planned to reach a sample of 1,000 adults from Basra, across a variety of social classes. I successfully gathered 715 full responses. To check whether participants took the survey seriously, I calculated the sample SD for answers of all questions for each individual response. I deleted 38 responses because the SD was close to zero –n i.e., the answers were the same for all questions. The final dataset comprised 677 completed responses. This process took three months, from August to October 2017. The dataset can be tracked and reused as shown in Appendix A. The supplementary data can be easily downloaded online at Al-Jundi [1].

**Fig. 1:**
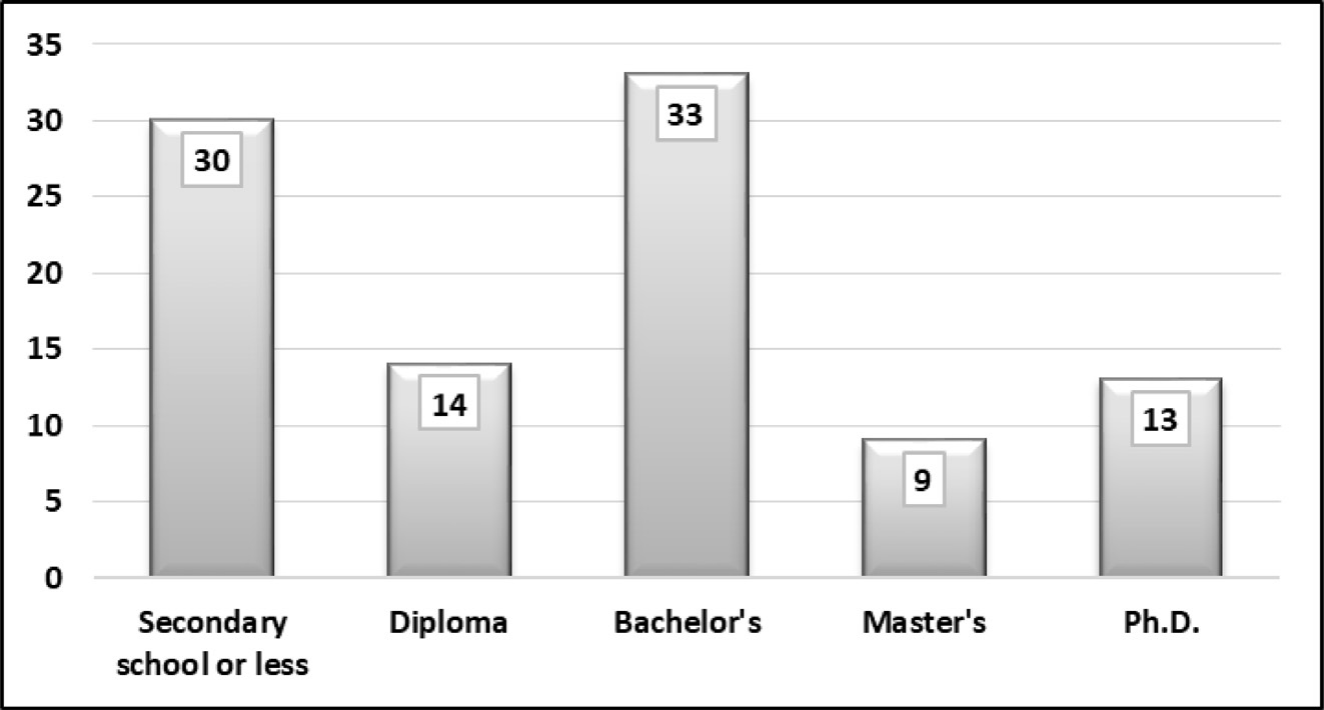
What is your highest educational level? (%).

**Fig. 2:**
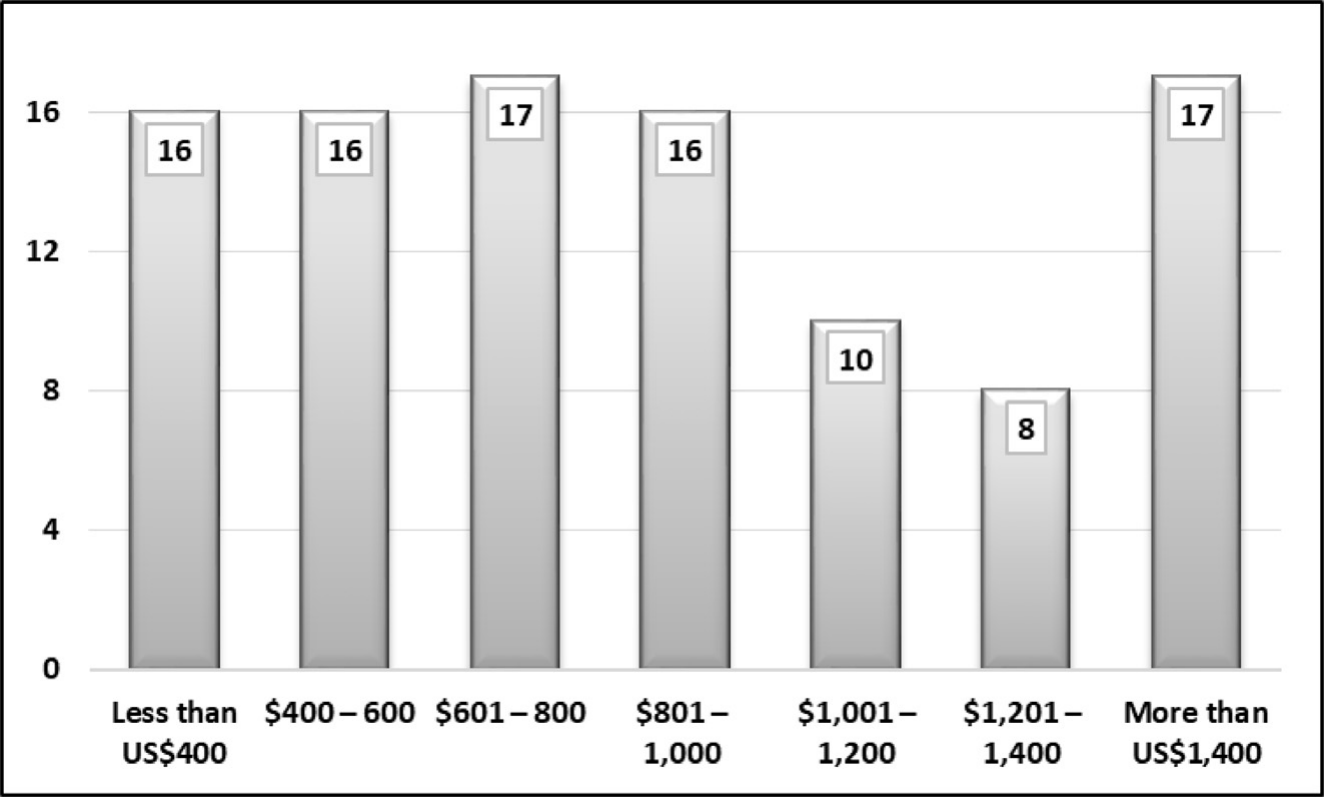
What is your monthly household income? (%).

**Fig. 3:**
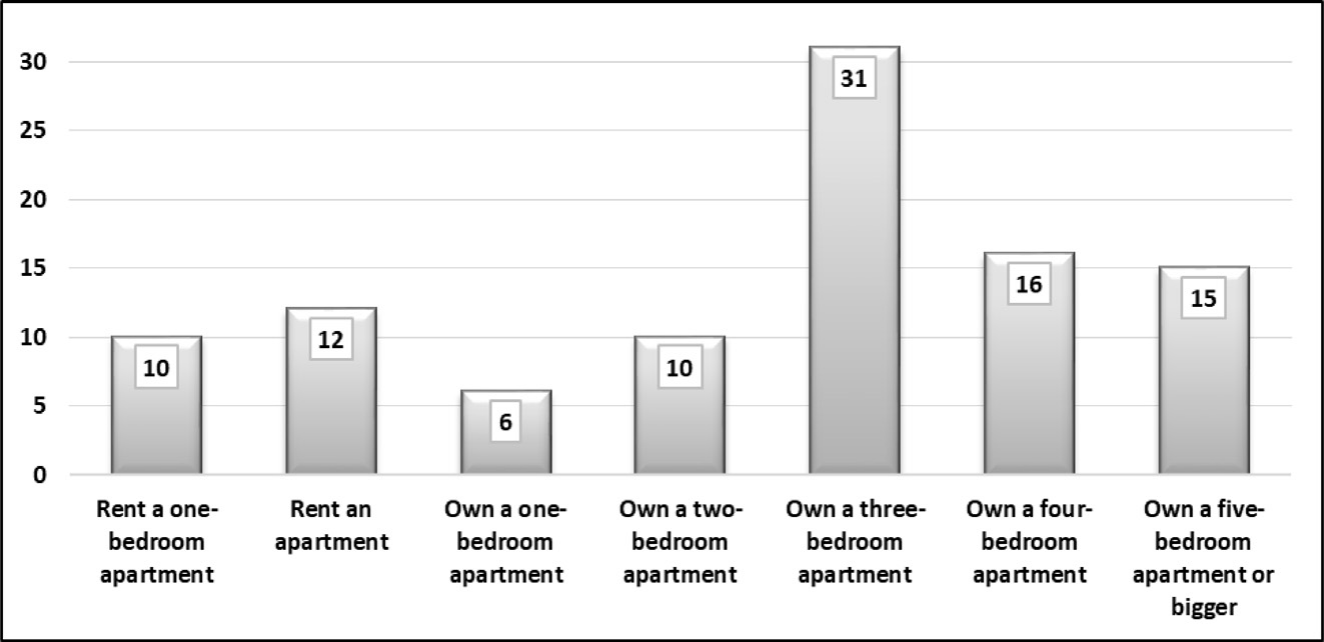
How big is your house? (%).

**Fig. 4:**
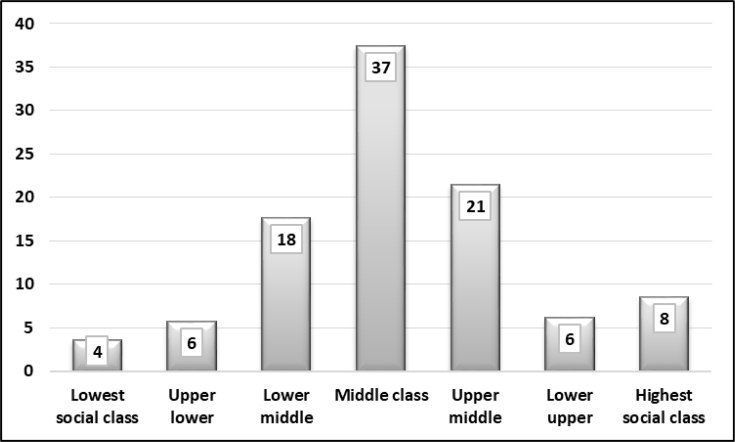
If we divide society into seven social strata, to which class do you belong? (%).

The sample represents different demographic groups in Basra. Fig. 1 shows that participants were mostly educated and had a bachelor's degree. People who were not educated or were poorly educated did not actively participate in the survey. Three per cent of the sample had an elementary school certificate or less. People who held secondary school certificates were mostly university students and did not yet hold a bachelor's degree. Fig. 2 displays the distribution of household monthly income. The sample was equal across different income levels. It revealed that the majority (65%) of respondents earned less than US$1,000 per month. Only one-third earned more than US$1,000. Fig. 3 demonstrates the style of housing in Iraq. Approximately one third of the sample owned a three-bedroom apartment and around other one-third owned larger than a three-bedroom apartment. This is the real style of housing in Iraq since rental apartments are somehow limited. Fig. 4 elucidates the perception of participants regarding the social classes to which they belong. It is a subjective assessment of social class [8]. The sample belonged primarily to the middle classes. The sample was thus quite diverse and fairly representative of the Basra community.

## Funding organizations

None.

## Conflict of Interest

The authors declare that they have no known competing financial interests or personal relationships that could have appeared to influence the work reported in this paper.
